# Two Case Reports of Varicocele Rupture during Sexual Intercourse and Review of the Literature

**DOI:** 10.1155/2018/4068174

**Published:** 2018-12-10

**Authors:** Chalil Arif, Konstantinos Kotoulas, Chrysostomos Georgellis, Konstantinos Frigkas, Athanasios Bantis, Emmanouil Patris

**Affiliations:** ^1^Department of Urology, University Hospital of Alexandroupolis, Greece; ^2^Department of Radiology, University Hospital of Alexandroupolis, Greece

## Abstract

Varicocele is characterized by the dilatation of the veins of the spermatic cord. Its prevalence in general male population is 15% while in the infertile population the prevalence rises up to 25%. The varicocele is considered an etiological factor for male infertility. Although different pathophysiological patterns have been proposed, there is no consensus in the urological society to date. In most of the cases varicocele is asymptomatic but sometimes gives mild symptoms as dull pain at the scrotal region. A rare complication of this condition is the spontaneous or traumatic rupture and hematoma formation, either as spermatic cord hematoma or as scrotal hematoma. We are presenting two cases of varicocele rupture, presented with acute painful swelling of the left inguinal and scrotal region during sexual intercourse. Imaging studies revealed a scrotal hematoma in the first case and a spermatic cord hematoma in the second case, without signs of active bleeding. Both patients were treated conservatively and recovered uneventfully. Subsequently, we reviewed the literature in an effort to find the key points for the diagnosis and treatment of this condition.

## 1. Introduction

Acute scrotal swelling is a potential emergency. A rare cause of scrotal swelling is the spontaneous or traumatic rupture of varicocele. Timely diagnosis of varicocele rupture is challenging as the symptoms are nonspecific and resemble other conditions such as torsion of the testis or one of the testicular appendages, hydrocele, trauma, tumor, idiopathic scrotal edema, and incarcerated inguinal hernia [1]. Although these conditions are rarely fatal, they may carry a risk of morbidity [2]. These two cases of varicocele rupture were diagnosed by ultrasound and MRI in the first case and only by ultrasound in the second. There was no active bleeding during the examination and therefore conservative therapy was administered.

## 2. Case 1

A 22-year-old male presented to the emergency department with painful swelling of the left hemiscrotum during sexual intercourse. The pain had sudden onset and was located at the left inguinal region. The patient noticed an extending swelling from the left inguinal area through the left hemiscrotum. The medical history revealed bilateral varicocele but no comorbidities or coagulation disorders. On examination, he had a painful swelling starting from the inguinal canal and extending through the entire left scrotum. A soft mass was palpable around the left testis and the spermatic cord. Laboratory tests were within normal range.

**Figure 1:**
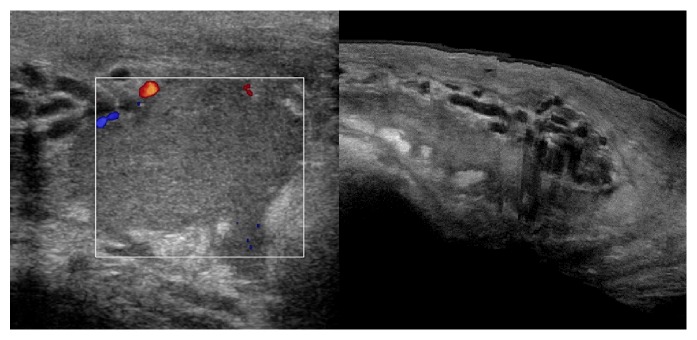
Doppler ultrasonography. Left: a well-defined area with heterogeneous echotexture on the left extratesticular tissue. No vascularity was noted in the lesion. Right: topography of the hematoma related to the varicocele. Scrotal wall thickening is also noted.

**Figure 2:**
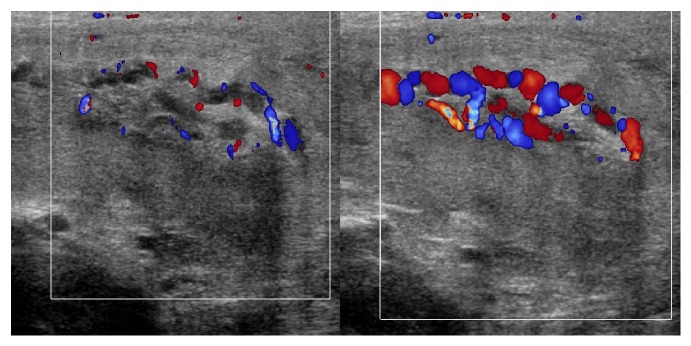
Doppler ultrasonography: dilatation of the pampiniform plexus of veins, image compatible with the varicocele.

**Figure 3:**
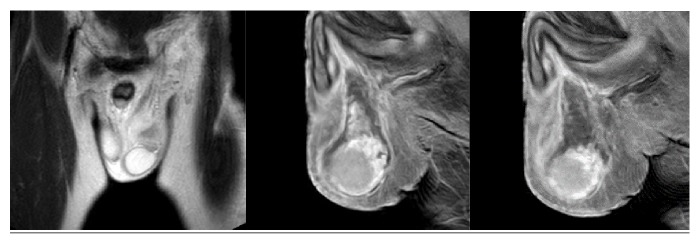
MRI of the scrotum which confirms the presence of hematoma. There is no contrast enhancement of the collection.

Doppler ultrasonography of the scrotum revealed a hematoma extending from the left superficial inguinal ring to the left hemiscrotum, partially surrounding the left testis. No active bleeding was observed (Figure 1). Both testes had normal structure and blood flow. The presence of bilateral varicocele was confirmed (Figure 2). Due to the rarity of the condition, an MRI of the scrotum was performed and confirmed the diagnosis of hematoma (Figure 3).

The patient was treated conservatively with bed rest, ice packs, antibiotics, and analgesics. During the hospitalization he was stable, the pain gradually decreased on the second day and the patient was discharged the third day. Ecchymosis of the scrotum was noticed at the first hospitalization day which gradually disappeared along with the swelling after 4 weeks. Bilateral varicocele repair was uneventfully performed three months later.

## 3. Case 2

A 24-year-old male presented to the emergency department due to left inguinal-scrotal pain which occurred acutely during sexual intercourse. The patient was otherwise healthy without comorbidities or hematological discrepancies. During the physical examination, a mild swelling of the left inguinal region expanding towards the left testis was observed. On palpation, a normal feeling testis but a thickened and painful spermatic cord was found. Laboratory tests were normal.

**Figure 4:**
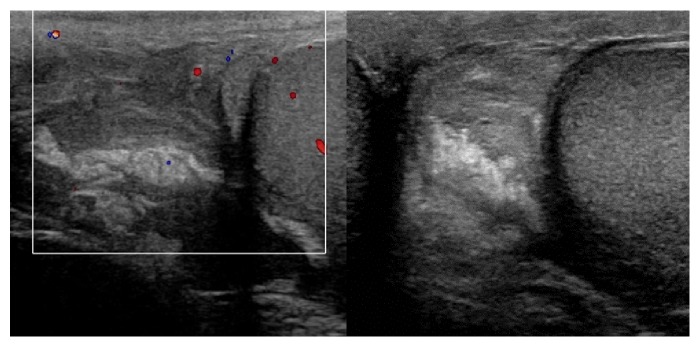
Doppler ultrasonography: a complex collection with internal echoes which is avascular on color Doppler, compatible with hematoma of the spermatic cord.

**Figure 5:**
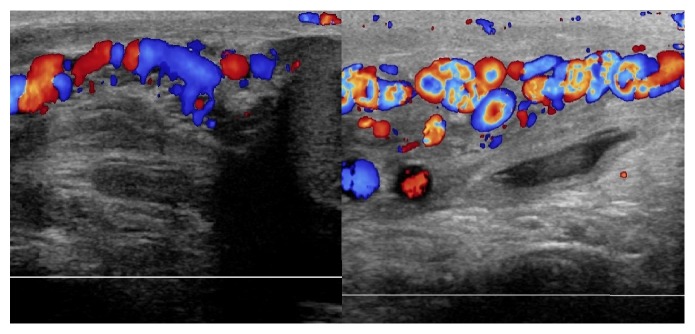
Doppler ultrasonography: dilated paratesticular veins, compatible with the presence of the varicocele. Presence of reflux during Valsalva maneuver.

Doppler ultrasonography of the scrotum revealed a spermatic cord hematoma expanding towards the upper pole of the left testis, with no active bleeding (Figure 4). Left varicocele was also observed (Figure 5). The patient refused hospitalization and MRI examination. He was discharged with instructions for bed rest, minimal physical activity, and prescription for analgesics and empirical antibiotic treatment. He returned to the emergency department 24 hours later with mild discomfort and ecchymosis of the left scrotum. A second ultrasound examination was performed showing a reduction of the hematoma and no additional abnormal findings. A follow-up examination was scheduled one week later, but the patient did not appear.

## 4. Discussion

The medical term varicocele describes the dilatation of the scrotal portion of pampiniform plexus and the internal spermatic venous system [3]. Its prevalence in general male population is 15%, which is significantly lower compared to the males with primary (35%) and secondary (80%) infertility [4]. Epidemiological data also indicate an increasing incidence of varicocele with age [5–7]. Oxidative stress, local hormonal imbalances, increased scrotal temperature, stasis of blood, and testicular hypoperfusion have been cited as possible mechanisms that affect spermatogenesis and the function of Leydig cells in men with varicocele, leading to infertility. Although different pathophysiological patterns have been proposed, there is no consensus in the urological society to date [8–13]. Chronic testicular pain is a common complaint, affecting up to 2% to 10% of patients with varicocele [14].

Spontaneous or traumatic rupture of the varicocele is a rare complication. Most commonly, causes of scrotal or spermatic cord hematoma are blunt trauma [15], Valsalva maneuver during defecation [16], Henoch-Schonlein syndrome [17], anticoagulant therapy [18], and lipomas [19] (Table 1). In our cases, the main cause was the spontaneous rupture of varicocele in otherwise healthy men, during sexual intercourse.

The clinical presentation of the cases reported in the literature varies considerably. In our cases the patients presented to the hospital immediately after the onset of the pain, while in other cases the patients seek medical care up to 7 days after the causative event [20]. The age of the patients also differs. The majority of the patients were at their mid-twenties, but a 78-year-old patient and a 69-year-old patient were also described [2, 21]. The side of the hematoma seems to be of importance, as in all of the reported cases the hematoma was left sided except in one case in which it was at the right hemiscrotum [2]. There is a clear correlation with the side of the varicocele which is usually (up to 80%) left sided [22].

The symptoms also varied considerably. In our cases, the first patient had severe pain and discomfort while the second presented with only mild pain and moderate discomfort. In the cases reported in the literature, patients experienced different degrees of pain, which in some cases was quite mild. The leading cause for referral was the scrotal swelling [23].

The clinical findings though were comparable. In the cases reported, as in our own experience, there was a noticeable enlargement of the hemiscrotum extending to the inguinal canal. In most cases there was a thickened spermatic cord and a palpable scrotal mass, but the consistency of the mass differed from fluctuant to firm [20, 24]. The testicle and the epididymis were normal on palpation in all cases. Ecchymosis of the scrotum was a common finding but most of the time appeared several hours after the clinical onset of the condition.

In our facility we routinely use Doppler ultrasonography to evaluate acute scrotal conditions. The same approach was used in the two cases. The examination showed normal blood flow to the testis, thus excluding ischemic conditions, successfully identifying the scrotal, spermatic cord hematoma. Bilateral varicocele was confirmed in the first case and left side varicocele was identified in the second. In the first case the diagnosis was confirmed the next day with an MRI study of pelvis and scrotum. Ultrasound of the retroperitoneal space was performed in both cases to exclude a retroperitoneal hemorrhage presenting as scrotal hematoma [25].

In most cases reported to date, imaging techniques were used to identify and to differentiate the cause of the acute scrotum. The Doppler ultrasonography was the most frequently used technique, although some physicians used abdominopelvic CT study [16, 24] and some used a combination of both [21]. In all cases the imaging techniques successfully detected the presence of the hematoma and the source of the bleeding in some cases [24, 26].

The type of treatment depends on patient's status, exclusion of the acute surgical conditions (i.e., testicular torsion and incarcerated hernia) and improvement of the symptoms. In our cases, considering that there was no absolute indication for surgical management, the treatment was conservative. Both patients were treated with NSAIDs, empiric antibiotics, bed rest, and ice packs, during and after hospitalization. The treatment strategies in the reported cases in literature were conservative when the clinical and imaging findings suggested scrotal hematoma [21, 24, 26], followed by uneventful recovery. In one case conservative treatment was unsuccessful and a scrotal exploration was performed five days later [23].

In cases where testicular torsion or incarcerated inguinal hernia was suspected or in presence of hemodynamic instability the first treatment option was surgery [2, 15, 16, 20, 25]. Both scrotal and inguinal approach was used, and evacuation of the hematoma and control of the bleeding vessels was performed.

## 5. Conclusion

Varicocele rupture is a rare complication of a common condition. High suspicion and careful evaluation of the patient is needed. Due to limited number of cases reported in the literature, it is difficult to draw a conclusion. With respect to the sparse evidence, some comments can be made. Scrotal hematoma is a difficult clinical diagnosis with no specific signs or symptoms. Imaging has an irreplaceable role in both differential diagnosis and identification of the spermatic cord, scrotal hematoma. Doppler ultrasonography seems to be sufficient in most cases, but it can be completed with a CT or MRI study if needed. The treatment depends on the patient's condition and the diagnostic certainty. The scrotal hematoma seems to be easily treated conservatively, but surgical exploration is the safest approach in case of uncertain diagnosis.

## Figures and Tables

**Table 1:** Various case reports.

	**Age**	**Side**	**Possible mechanism**	**Time to presentation**	**Clinical findings**	**Radiological study findings**	**Treatment**
Chin et al. [23]	33	Left	Lifting a heavy piece of furniture	2 days	Markedly swollen and thickened left hemiscrotum with the swelling extending up to the left inguinal canal	Ultrasonography: Large left scrotal hematoma	Scrotal exploration.

Matsui et al. [21]	69	Left	Straining for defecation	10 days	Distended mass extending inferiorly through the left groin region and into the left hemiscrotum	Computed tomography: Dilated spermatic cord with large scrotal hematoma	Managed conservatively

Nishiyama et al. [26]	23	Left	Sexual intercourse	Same day	Ecchymosis and swelling of the left hemiscrotum	Ultrasonography: Blood flowing into the space surrounding the left testis and hematoma formation	Managed conservatively

Aliabadi et al. [16]	27	Left	Straining for defecation	One hour	Swelling and tenderness over the inguinal canal		Scrotal exploration

Gordon et al. [15]	22	Left	Struck the abdomen against the handle bars of the motorcycle	Immediately	Tender fullness of the left inguinal region	Computed tomography: Enlarged left spermatic cord	Scrotal exploration

Lerman et al. [20]	23	Left	Pulling on a hand wrench	Immediately	10 cm tender and firm mass palpated in the left hemiscrotum.		Scrotal exploration

Lerman et al. [20]	21	Left	Moving machinery	7 days	Left hemiscrotum was 1 1/2 times larger than normal and the overlying skin was ecchymotic		Scrotal exploration

Burnard et al. [2]	78	Right	Unknown	12 hours	Expanding tense scrotal hematoma Significant drop in hemoglobin levels Signs of hemorrhagic shock	Ultrasonography: Large scrotal hematoma Computed tomography: Large right-sided hematocele of mixed attenuation consistent with acute hemorrhage and evidence of extravasation of contrast at the superior pole of the hematocele	Scrotal exploration Inguinal orchiectomy
